# Recent Advances in Fragment-Based QSAR and Multi-Dimensional QSAR Methods

**DOI:** 10.3390/ijms11103846

**Published:** 2010-10-08

**Authors:** Kyaw Zeyar Myint, Xiang-Qun Xie

**Affiliations:** 1 Department of Computational Biology, School of Medicine, University of Pittsburgh, Pittsburgh, PA 15260, USA; E-Mail: kym3@pitt.edu; 2 Department of Pharmaceutical Sciences, School of Pharmacy, University of Pittsburgh, Pittsburgh, PA 15260, USA; 3 Pittsburgh Chemical Methodologies & Library Development (PCMLD) and Pittsburgh Drug Discovery Institute, University of Pittsburgh, Pittsburgh, PA 15260, USA

**Keywords:** QSAR, fragment similarity based, fragment-based, 2D-QSAR, 3D-QSAR, nD-QSAR

## Abstract

This paper provides an overview of recently developed two dimensional (2D) fragment-based QSAR methods as well as other multi-dimensional approaches. In particular, we present recent fragment-based QSAR methods such as fragment-similarity-based QSAR (FS-QSAR), fragment-based QSAR (FB-QSAR), Hologram QSAR (HQSAR), and top priority fragment QSAR in addition to 3D- and nD-QSAR methods such as comparative molecular field analysis (CoMFA), comparative molecular similarity analysis (CoMSIA), Topomer CoMFA, self-organizing molecular field analysis (SOMFA), comparative molecular moment analysis (COMMA), autocorrelation of molecular surfaces properties (AMSP), weighted holistic invariant molecular (WHIM) descriptor-based QSAR (WHIM), grid-independent descriptors (GRIND)-based QSAR, 4D-QSAR, 5D-QSAR and 6D-QSAR methods.

## 1. Introduction

Quantitative structure-activity relationship (QSAR) is based on the general principle of medicinal chemistry that the biological activity of a ligand or compound is related to its molecular structure or properties, and structurally similar molecules may have similar biological activities [1]. Such molecular structural information is encoded in molecular descriptors and a QSAR model defines mathematical relationships between descriptors and biological activities of known ligands to predict unknown ligands’ activities. QSAR methods have been applied in several scientific studies including chemistry, biology, toxicology and drug discovery to predict and classify biological activities of virtual or newly-synthesized compounds [2–6]. QSAR models can also be used in designing new chemical entities (NCEs) and are now regarded as essential tools in pharmaceutical industries to identify promising hits and generate high quality leads in the early stages of drug discovery [5,7]. In other words, QSAR studies can reduce the costly failures of drug candidates by identifying the most promising hit compounds and reducing the number of costly experiments.

**Figure 1:**
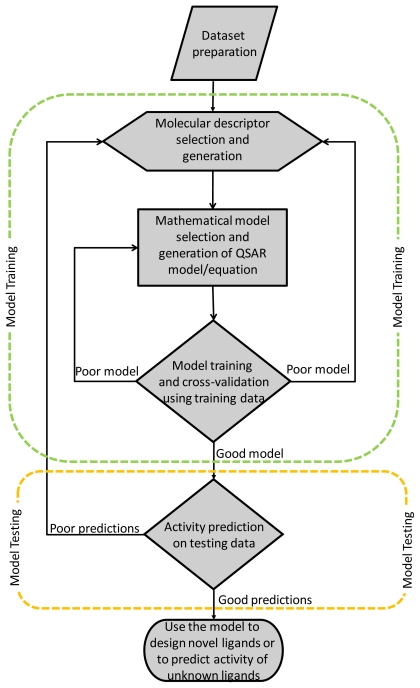
A general scheme of a QSAR model development which includes systematic training and testing processes.

In general, QSAR modeling (Figure 1) involves a systematic process with multiple steps, including dataset preparation, molecular descriptors selection and generation, mathematical or statistical models derivation, model training and validation using a training dataset and model testing on a testing dataset. During the first step, or dataset preparation, it is important to pay attention to the quality of data to develop a reliable QSAR model. Data should come from the same bioassay protocols and it is preferable to collect and use the data generated from a single lab or source in order to avoid data inconsistencies and interlaboratory variability. Moreover, the dataset should have a large enough number of compounds to ensure statistical stability of a QSAR model and the bioactivity should cover a range of values with a good distribution [5]. The second step in QSAR modeling is the selection and generation of molecular descriptors for ligands in the dataset. There are many descriptors available and only some of them are significantly correlated with the activity. Therefore, selection of appropriate descriptors, which best capture the structural variation and information is important to derive a robust QSAR model. Several methods such as evolutionary algorithms (for example, genetic algorithm) and machine learning techniques (for example, forward selection) can be used for descriptor/variable/feature selection. After molecular descriptors are defined and generated for all ligands in the dataset, the next step is to decide a suitable statistical or mathematical model to find the relationship between such descriptors and biological activities. For instance, linear approaches such as multiple linear regression (MLR) or partial least square (PLS) and non-linear methods such as neural networks or support vector machine can be used as correlation or mapping functions. Once a model is chosen, it is then trained on a training dataset which contains a subset of randomly selected compounds from a known dataset, leaving the remaining to be used as testing compounds. During the model training, validation methods such as leave-one-out cross-validation (LOOCV) are often performed to ensure the statistical stability of the QSAR model. The training process is repeated until a satisfactory training performance is achieved. Finally, a testing process is performed in which the trained model is used to predict activity values of those compounds in the testing set.

A wide range of QSAR methodologies have been invented since the concept was first introduced by Free, Wilson, Hansch, and Fujita [8,9] in 1964. Traditional 2D-QSAR methods such as Free-Wilson and Hansch-Fujita models use 2D molecular substituents or fragments and their physicochemical properties to perform quantitative predictions. Since then, QSAR has experienced a fast development and the first novel 3D-QSAR method called comparative molecular field analysis (CoMFA) was introduced by Cramer *et al.* in 1988. The CoMFA method brought a foundation for the development of other 3D-QSAR methods such as CoMSIA, SOMFA, CoMMA as well as multidimensional (nD)-QSAR methods such as 4D-QSAR, 5D-QSAR, *etc*., to tackle known 3D-QSAR problems such as subjective molecular alignment and bioactive conformation problems. In recent years, fragment-based methods have attracted some attention because predicting molecular properties and activities based on molecular fragments is simple, fast and robust. In this review, we present recently available fragment-based QSAR methods and multidimensional (nD)-QSAR methods developed over the past few decades.

## 2. Fragment-Based 2D-QSAR Methods

Over the years, improved methods—that are based on such traditional QSAR methods—have been introduced. 2D methods allow modeling of a wide variety of ligands or compounds including cases where 3D crystal receptor or target structures are not available [7].

### 2.1. Hologram-QSAR (HQSAR)

**Figure 2:**
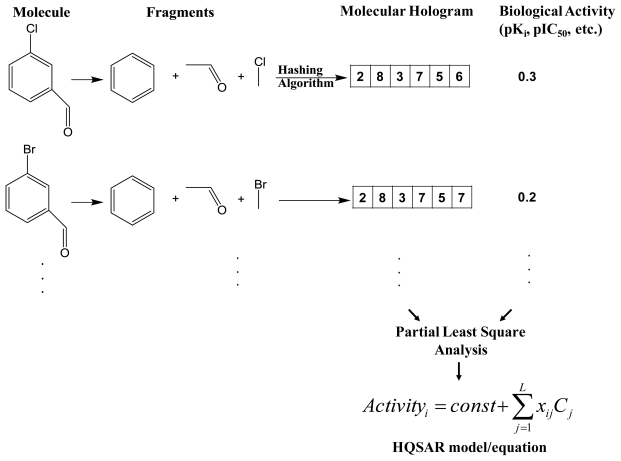
Hologram-QSAR (HQSAR) model development, which includes molecular hologram generation and partial least square analysis to derive a final predictive HQSAR equation.

One earlier example of a fragment-based method is HQSAR (Hologram QSAR) from Tripos [10,11]. Given a method based on 2D molecular fragments, HQSAR does not require molecular alignment and therefore allows for automated analyses of large data sets without manual intervention. The first step in the HQSAR method is to generate molecular holograms which contain counts of molecular fragments and can be related to 2D fingerprints. As depicted in Figure 2, the input dataset contains 2D structures of compounds and they are split into all possible linear and branched fragments. Then each unique fragment is assigned to a specific large positive integer by using a cyclic redundancy check (CRC) algorithm. All fragments generated are then hashed into array (hologram) bins in the range from 1 to L (total length of hologram). Bin occupancies represent counts of fragments in each bin. In other words, they are structural descriptors, which contain topological and compositional molecular information. During the second step, such fragment counts or hologram bins are correlated to corresponding biological activities (dependent variables) in a form of mathematical equation. Leave-one- out cross-validation (LOOCV) is performed to identify an optimal number of explanatory variables or components which yields an optimal model. Then by using standard partial least square (PLS) analysis, a following mathematical regression equation is derived to correlate hologram bin values or components with corresponding biological activities:

(1) $${BA}_{i}=const+\sum_{j=1}^{L}x_{ij}C_{j}$$

where *BA_i_* is the biological activity of the i^th^ compound, *x_ij_* is the occupancy value of the molecular hologram of the i^th^ compound at position or bin *j*, *C_j_* is the coefficient for the bin *j* derived from the PLS analysis, and *L* is the length of the hologram.

One drawback of HQSAR is a phenomenon called a fragment collision problem which happens during the hashing process of fragments. Although hashing reduces the length of the hologram, it causes bins to have different fragments in the same bin. The hologram length, a user-definable parameter, controls the number of bins in the hologram and alteration of hologram length can causes the pattern of bin occupancies to change. The program provides 12 default lengths which have been found to give good predictive models on different datasets. Each of these default lengths provides a unique set of fragment collisions [11].

Several HQSAR models for different ligand datasets including cases where the 3D crystal structure of receptor targets or proteins are unavailable have been developed in recent years [12–15]. For example, HQSAR was used to study a set of 9-substituted-9-deazaguanine analogs which inhibit the human purine nucleoside phosphorylase (PNP) enzyme. HQSAR was used to identify structural features with poor and favorable contributions towards molecular interactions in the active site [12]. In addition, HQSAR has been used in virtual screening to identify hits [16–18]. For instance, Salum *et al.* studied a set of 180 indole derivatives having potent anticancer activity. They developed several HQSAR models and compared them to determine optimal cutoff values in virtual screening procedures [7].

### 2.2. Fragment-Based QSAR (FB-QSAR)

Recently, Du *et al.* [19] introduced a 2D-QSAR method based on molecular fragments. The method uses a mixed Hansch-Fujita [9] linear free energy equation and Free-Wilson [8] equation. In particular, molecular fragments are first generated from ligands and the total binding free energy Δ*G_i_^o^* between ligand *i* and the receptor is considered as the sum of contributions Δ*g_i,α_* from all fragments:

(2) $$\Delta G_{i}^{o}=\sum_{\alpha =1}^{M}b_{\alpha }\Delta g_{i,\alpha }$$

where Δ*g_i,α_* is the free energy contribution of fragment *F_i,α_* and *b_α_* is a weight coefficient for each fragment. The binding free energy of a fragment, Δ*g_i,α_*, is described by a set of physical and chemical properties of the fragment:

(3) $$\Delta g_{i,\alpha }=\sum_{l=1}^{L}a_{l}p_{i,\alpha ,l}$$

where *p_i,α,l_* is the *l*-th property of fragment *F_i,α_* in molecule *m_i_* and *a_l_* is the coefficient of *l*-th property of the fragment.

In their studies, a total of 48 neuraminidase (NA) inhibitor analogs were used to train and test the model. Ten physicochemical properties were calculated for each substituent. Using an iterative double least square (IDLS) procedure, two sets of coefficients, one for fragments (*b_α_* from Equation 2) and another for physicochemical properties (*a_l_* from Equation 3), in the linear equation were solved alternately and iteratively until the model met the convergence criterion. After 176 iterations, the model converged and both sets of coefficients were solved. Such converged coefficients were used for the test calculation and the correlation coefficient (r) was 0.9525 (or r^2^ = 0.91). They also tested on Free-Wilson and Hansch-Fujita models, which achieved r values of 0.2488 (r^2^ = 0.06) and 0.9373 (r^2^ = 0.88), respectively. The quantitative results proved the IDLS procedure enhanced the predictive power, and, given a novel method, more applications are necessary to fully explore its predictive potential.

### 2.3. Fragment-Similarity Based QSAR (FS-QSAR)

More recently, a fragment-similarity based QSAR (FS-QSAR) method [20] was developed to solve the major limitation of the original Free-Wilson method by introducing the fragment-similarity concept in the linear regression equation. Such a similarity concept was applied for the first time to improve the traditional Free-Wilson equation instead of using physicochemical properties which often produce non-unique solutions. In this approach, the fragment similarity calculation was carried out by the similarity. It used the lowest or highest eigen values calculated from BCUT-matrices [21,22], which contained partial charges of individual atoms and their atomic connection information in each individual fragments. The updated equation of the FS-QSAR is as follows:

(4) $$-\text{log}K_{i}=const+\sum_{j=1}^{N}[\text{max}\{\overset{P_{j}}{\underset{k=1}{Sim}}(F_{jk},F_{jg})\}]\times A_{j}^{MSF}$$

*N* = the total number of substituent positions.

*P_j_* = the total number of possible substituents at the j^th^ substituent position.

max = the max function picks the maximum score among similarity scores.

*F_jk_* = the k^th^ fragment (a known fragment in the training set) at the j^th^ substituent position.

*F_jg_* *=* a given fragment (the fragment from a testing/unknown compound) at the j^th^ substituent position.

*Sim[F_jk,_* *F_jg_]* = the fragment similarity function compares *F_jg_* to *F_jk_* and calculates a similarity score.

*A_j_^MSF^* = the coefficient of the most similar fragment (MSF) at the j^th^ substituent position.

The similarity function used in Equation (4) is defined as:

(5) $$Sim(F_{jk},F_{jg})=e^{\left|EV(F_{jk})-EV(F_{jg})\right|}$$

where EV(F_jk_) = lowest or highest eigen value of BCUT matrix of a fragment (F_jk_).

The algorithm was developed and then tested on different datasets including 83 COX2 analogs and 85 triaryl bis-sulfone analogs. For statistical modeling, the model was repeatedly tested on five different testing sets which were generated by random selection of compounds. The average squared correlation coefficient, r^2^, over five testing sets was 0.62 for COX2 analogs and 0.68 for bis-sulfone analogs. For comparison, the original Free-Wilson method was also tested, achieving the average r^2^ values of 0.46 for COX2 dataset and 0.42 for bis-sulfone dataset. Moreover, for better comparison the BCUT-similarity function was replaced by Tanimoto coefficient (Tc) method, the traditional 2D molecular similarity function, and the average r^2^ was 0.62 for both COX2 and bis-sulfone analogs. The FS-QSAR method was proved to have an effective predictive power compared to the traditional 2D-QSAR method since it solved the major limitation of the original Free-Wilson method by introducing the similarity concept into the regression equation. However, the predictive accuracy of FS-QSAR may not be as high as other higher dimension QSAR methods, but the method provides an objective, unique and reproducible 2D-QSAR model.

### 2.4. Top Priority Fragment QSAR

Casalegno *et al.* [23] introduced a fragment-based QSAR approach to predict pesticide aquatic toxicity to the rainbow trout. The method prioritizes fragments’ contributions to toxicity with the assumption that one fragment among others present in a compound is mainly responsible for the toxicity. They used 282 carefully selected pesticides which were partitioned into 240 training and 42 testing molecules. In the first stage, all 282 molecules were broken into small substructures or atomic centered units (ACUs). Then, a numerical criterion based on the training set toxicity data was applied to assign one fragment or top-priority fragment (TPF), made up of one or more ACUs, to each training molecule. Once the TPFs were extracted, a ‘priority matrix’ was used to extract all priority relationships. A priority matrix contains information among training TPFs and can be used to find out which TPF has a priority to be assigned to a testing molecule. In the last stage, testing molecules were submitted to check for the presence of TPFs and information from the priority matrix was used to identify the ones(s) with highest priority, and final prediction was made based on average fragment toxicity. The final r^2^ for the training set was 0.85 and 0.75 for the test set proving the model’s effectiveness.

### 2.5. Other Fragment-Related QSAR Studies

In recent years, some new fragment-based QSAR methods have been discovered as well as applications to biological interests. Zhokhova *et al.* [24] introduced a method which uses fragmental descriptors with labeled atoms and applied it to their QSAR/QSPR (quantitative structure-property relationship) studies. In their approach, the fast stepwise multiple linear regression (FSMLR) and three-layer artificial neural network (ANN) methods implemented in the NASAWIN program [25] were used to generate fragmental descriptors with labeled atoms and to construct QSAR/QSPR models. Andrade *et al.* [26] used HQSAR and other 2D-QSAR programs to study a series of hydrazides as antituberculosis agents. They used DRAGON 5.4 [27], BuildQSAR [28], PIROUETTE [29] programs for generation and selection of 2D molecular descriptors. Tsygankova *et al.* [30] also did the QSAR studies of barbituric acid derivatives using 2D fragments as descriptors with different regression approaches such as step-by-step regression to construct correlation equations.

## 3. 3D-QSAR

The 3D-QSAR methods have been developed to improve the prediction accuracies of 2D methods. 3D methods are computationally more complex and demanding than 2D approaches. In general, there are two families of 3D-QSAR methods: alignment-dependent methods and alignment-independent methods. Both families need experimentally or computationally derived bioactive conformations of ligands as templates for studies. Such 3D conformers are one of the most important factors to produce reliable 3D-QSAR models and are also the major drawbacks of 3D methods. Examples of both families are discussed below.

### 3.1. Comparative Molecular Field Analysis (CoMFA) and Comparative Molecular Similarity Indices Analysis (CoMSIA)

**Figure 3:**
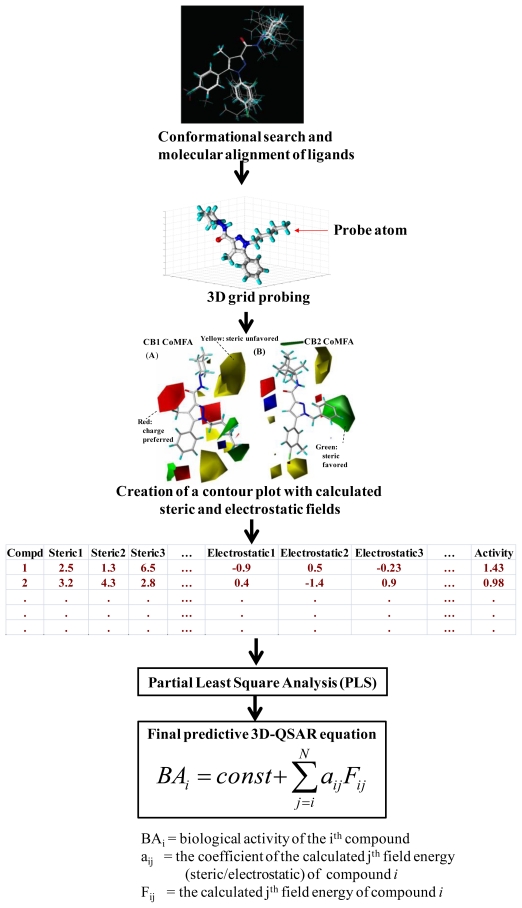
A general CoMFA workflow.

One of well-known methods is a three dimensional QSAR method called CoMFA developed by Cramer *et al.* [31]. It is a method to describe 3D structure-activity relationship quantitatively by considering 3D structures, and steric and electrostatic fields of ligands which are superimposed to generate such molecular fields. In other words, CoMFA is an alignment-dependent method in which molecular field interaction energy terms are correlated with biological activities/responses using multivariate statistical analyses. Figure 3 illustrates a general CoMFA modeling process where active molecules are first placed in a 3D grid. Using a probe atom, steric and electrostatic energies are measured at each grid point for each molecule. Partial least square (PLS) analysis is then performed to correlate such field energy terms to activity values and make predictions. Such features and calculations make CoMFA an improved and different method from other traditional QSAR approaches.

Another 3D QSAR method named CoMSIA by Klebe *et al.* is similar to CoMFA in terms of using a probe atom along grid points. However, additional molecular fields have been implemented in the CoMSIA approach. In particular, electrostatic, steric, hydrophobic, hydrogen bond acceptor (HBA), and hydrogen bond donor (HBD) properties are generated using a Gaussian distance function [32]. Using such a Guassian-type potential function instead of Lennard-Jones and Coulombic functions provides accurate information at grid points for calculating molecular fields [33].

However, the major drawback of both methods is that all molecules have to be aligned and such alignment can affect the final CoMFA/CoMSIA model and predictions. A good alignment is necessary and quality of such alignment can be subjective, time-consuming [34] and CoMFA/CoMSIA models are sometimes non-reproducible [33]. Nevertheless, several CoMFA/CoMSIA models have been developed for many drug design and molecular modeling studies [6,35–39]

### 3.2. Topomer CoMFA

Recently, Cramer *et al.* introduced a new QSAR method named the Topomer CoMFA [40] which is a rapid fragment-based 3D-QSAR method to predict significant R-groups, which can optimize the biological activities as well as optimized structural changes for lead scaffold hopping. It uses the compound library collection as a source of molecular fragments to identify such substituents or R-groups. The Topomer CoMFA method, unlike CoMFA, does not require the subjective alignment of 3D ligand conformers and uses automated alignment rules. A topomer describes both a conformation and orientation of a molecular fragment and it is generated based on 2D structure without any relation to a receptor site or other ligands [34,40]. After such topomers are generated, CoMFA analysis is then carried out where electrostatic and steric fields are calculated using a probe atom around the 3D grid. Subsequently, partial least square (PLS) with leave-one-out cross-validation is performed to generate a predictive model. 15 3D-QSAR analyses retrieved from the literature yielded an average q^2^ of 0.520 compared to literature average q^2^ of 0.636 [40]. Topomer CoMFA has the potential to optimize biological activities of ligands via fragments and has been used in lead-optimization and R-groups virtual screening studies [34,40]

### 3.3. Self-Organizing Molecular Field Analysis (SOMFA)

Robinson *et al.* [41] introduced another alignment-dependent 3D-QSAR method called SOMFA, which is based on both molecular shape and electrostatic potentials. Briefly, 3D grids are created as in other 3D-QSAR methods and for each grid point, molecular shape and electrostatic potential values are calculated. Shape values are binary meaning 1 for being inside the van der Waals envelope and 0 outside. The key step is that the electrostatic potential value at each grid point is multiplied by the mean centered activity for that molecule as a weighing factor which causes the most active and least active molecules to have higher values than other common and less interesting molecules which are closer to the mean activity. The SOMFA grid value at a given *x*,*y*,*z* is defined as:

(6) $${\text{SOMFA}}_{\text{x},\text{y},\text{z}}=\sum_{\text{i}}^{\text{Training Set}}{\text{Property}}_{\text{i}}(\text{x},\text{y},\text{z})\text{Mean}\_\text{Centered}\_\text{Activity}$$

Using such a property master grid, an estimate of the activity of the i^th^ molecule as defined by a certain property can be derived as:

(7) $${\text{SOMFA}}_{\text{property},\text{i}}=\sum_{\text{x}}\sum_{\text{y}}\sum_{\text{z}}{\text{Property}}_{\text{i}}(\text{x},\text{y},\text{z}){\text{SOMFA}}_{\text{x},\text{y},\text{z}}$$

In the final stage, correlations between calculated SOMFA property values (SOMFA_property, i_) and biological activities are derived via multiple linear regression and a final predictive model is produced. Robinson *et al.* tested the model using two datasets: 31 steroid compounds and 35 sulfonamides. The corresponding correlation coefficient values (r^2^) of 0.5776 (r = 0.76) and 0.5329 (r = 0.73) were achieved, respectively. Compared to other methods such as CoMFA [31], MS-WHIM [42] and few others on steroid dataset, SOMFA had the lowest standard deviation of errors of prediction (SDEP), which is the root-mean-square error of the predictions. In short, SOMFA is similar to CoMFA in terms of using grids and necessity of molecular alignment but is not as statistically rigorous as CoMFA [1], as the SOMFA model is conceptually simple without heavy statistical elements such as partial least square (PLS).

### 3.4. Alignment-Free 3D-QSAR Methods

In the last few decades, other 3D-QSAR methods which do not rely on alignments were introduced. Some examples include autocorrelation of molecular surfaces properties (AMSP) [43], comparative molecular moment analysis (CoMMA) [44], WHIM (Weighted Holistic Invariant Molecular) method [45,46], Molecular surface (MS)-WHIM [42], and GRIND [47].

#### 3.4.1. Autocorrelation of Molecular Surfaces Properties (AMSP)

Wagener *et al.* introduced the AMSP method to map the physical properties of ligands to a van der Waals surface and individual atoms, respectively. It uses a 3D descriptor based on spatial autocorrelation of molecular properties at distinct points on the molecular surface. The points are randomly distributed to have a continuous surface and the autocorrelation coefficient is obtained by summing the products of property values at various pairs of points at particular distances. For a series of distance intervals (d_lower_, d_upper_), a vector of autocorrelation coefficients is obtained as follows:

(8) $$A(d_{lower},d_{upper})=\frac{1}{L}\sum_{ij}p_{i}p_{j}\;\;\;(d_{lower}<d_{ij}<d_{upper})$$

where *p_i_* is the molecular property value at point *i*, *p_j_* is the molecular property value at point *j* and L is the total number of distances in the interval [43].

Therefore, the vector contains a compressed expression of the distribution of a property on the molecular surface. After autocorrelation vectors were obtained, a multilayer neural network was then trained using such vectors to derive a predictive model of biological activity of 31 steroid compounds. The correlation coefficient value, r, of 0.82 (r^2^ = 0.6724) was achieved with a cross-validated r^2^ of 0.63. In summary, the advantages of such autocorrelation vectors are the facts that they are shown to be invariant to translation and rotation since only spatial distances are used and have condensed description of molecular surface. However, original information cannot be reconstructed from such condensed vectors and the pharmacophore nature of a ligand may not be clear or interpretable [43].

#### 3.4.2. Comparative Molecular Moment Analysis (CoMMA)

Silverman *et al.* [44] introduced the CoMMA method, which calculates the zeroth-, first-, and second-order spatial moments of the charge (such as quadrupolar moments) and the mass distribution (such as moments of inertia). Such molecular moment descriptors may be classified in three different categories: descriptors relating solely to molecular shape, descriptors relating only to molecular charge and descriptors relating to both shape and charge. The authors calculated 13 such descriptors and used them in partial least square analysis to generate predictive QSAR models for 31 steroid compounds. A range of statistical performance was obtained depending on different partial charge calculation methods used to derive electrostatic moments. Cross-validated r^2^ values ranging from 0.412 to 0.828 were obtained using electrostatic moment descriptors calculated from Gasteiger charges or Guassian molecular orbital *ab initio* methods. The results showed that using quantum chemistry calculation-based moments produced better predictive models than using only Gasteiger charge-based moments. Despite CoMMA’s comparable statistical performances to CoMFA’s, there are some limitations which may account for the limited number of published CoMMA applications. One reason is that the value of these descriptors, which measures the displacement between the center of mass and center of dipole with respect to the principal inertial axes, equals infinity for symmetric molecules whose dipole moment is zero [5].

#### 3.4.3. Weighted Holistic Invariant Molecular (WHIM) Descriptor-Based QSAR

WHIM descriptors contain 3D molecular information such as molecular size, shape, symmetry and distribution of molecular surface point coordinates [45,46]. Molecular surface (MS)-WHIM is a WHIM-based 3D descriptor derived directly from molecular surface properties [42]. For WHIM descriptors, two types of matrices are defined: a molecular matrix containing cartesian coordinates of the *n* atoms and diagonal matrices containing the weights which are physicochemical properties associated with the *n* atoms of the molecule [42]. Each element of the diagonal matrix is defined as:

(9) $${\text{S}}_{\text{jk}}=\frac{\sum_{\text{i}=1}^{\text{n}}{\text{W}}_{\text{i}}\left({\text{q}}_{\text{ij}}-\bar{{\text{q}}_{\text{j}}}\right)\left({\text{q}}_{\text{ik}}-\bar{{\text{q}}_{\text{k}}}\right)}{\sum_{\text{i}-1}^{\text{n}}{\text{W}}_{\text{i}}}$$

where *n* is the number of atoms, w_i_ is the weight of ith atom, *q_ij_* is the *j*^th^ coordinate of the *i*^th^ atom and $\bar{{\text{q}}_{\text{j}}}$ is the average of the *j*^th^ coordinates [45].

In this expression, atoms can be weighted by mass, van der Waals volume, atomic electronegativity, electrotopological index of Kier and Hall, atomic polarizability and molecular electrostatic potential [33]. Elements in each diagonal matrix are subjected to principal component analysis (PCA) to obtain the scoring matrix, which is used to calculate PCA eigen values and eigen value proportion. Such values and proportions are then correlated with the molecular size and shape, respectively. One major advantage of the WHIM approach is that it provides a 3D QSAR descriptor which is invariant to translation and rotation of 3D molecular structures. In MS-WHIM, properties associated with the molecular surface points are used as different weighting schemes to compute statistical parameters. In particular, the unitary value and molecular electrostatic potential (MEP) are computed at each point of the Connolly molecular surface [48], and they are considered as weights. The unitary value contains information about the molecular surface shape and MEP provides the electrostatic information about the electron density distribution [42]. Although the WHIM approach is not sensitive to molecular orientation, MS-WHIM descriptor values are affected by the facts that the Connolly surface points are dependent on the 3D orientation of the molecule and indices for different weighting schemes are sensitive to surface point density [42]. The authors tested both WHIM and MS-WHIM on 31 steroid compounds and achieved the SDEP (standard deviation error of prediction) values of 1.750 and 0.742, respectively while CoMFA’s SDEP was 0.837. The results suggested that MS-WHIM prediction performance was comparable to CoMFA’s. SDEP was defined as follows:

(10) $$\text{SDEP}=\sqrt{\frac{\sum {\left({\text{y}}_{\text{pred}}-{\text{y}}_{\text{obs}}\right)}^{2}}{\text{n}}}$$

WHIM/MS-WHIM descriptors are invariant to 3D molecular orientation but both methods, like other 3D-QSAR methods, rely on ligand conformation, which may be subjective if ligand-receptor co-crystal structures are not known for the target of interest.

#### 3.4.4. Grid-Independent Descriptors (GRIND)-Based QSAR

In an attempt to provide alignment-free descriptors which are easy to understand and interpret, Pastor *et al.* introduced grid-independent descriptors [47]. The method utilizes specific probes such as the O probe (carbonyl oxygen) and N1 probe (amide nitrogen) to calculate molecular interaction fields (MIFs) at grid points. At each node of the grid, the energy between the probe and target ligand (E) is calculated as:

(11) $$\text{E}=\sum {\text{E}}_{\text{es}}+\sum {\text{E}}_{\text{hb}}+\sum {\text{E}}_{1\text{j}}$$

where E_es_ is the electrostatic energy, E_hb_ is the hydrogen-bonding energy, and E_lj_ is the Lennard-Jones potential energy [49].

In this method, electrostatic interactions, hydrophobic interactions, hydrogen bond acceptor and hydrogen bond donor fields are considered to get a set of positions which defines a ‘virtual receptor site’ (VRS). VRS regions are then encoded into GRIND via an auto- and cross-correlation transform so that those regions are no longer dependent upon their positions in the 3D space. In other words, autocorrelation descriptors of the fields are calculated and only the highest products of molecular interaction energies are stored while others are discarded. This difference is responsible for the ‘reversibility’ of GRIND and the descriptors can be back-projected in 3D space using another related program called ALMOND [50]. The statistical performance of GRIND is comparable to other methods, but the advantage is that it is alignment-free and easy to interpret. However, bioactive conformations of ligands are valuable information to derive the virtual receptor site (VRS) and limitations on such information may affect final predictive models like other 3D methods.

### 3.5. Multi-Dimensional (nD) QSAR Methods

Multi-dimensional (nD) QSAR methods are essentially extensions of 3D-QSAR methods. These methods incorporate additional physical characteristics or properties (or a new dimension) to tackle the drawbacks of 3D-QSAR methods. One example is 4D-QSAR by Hopfinger *et al.* [51] which samples molecular conformations and alignments during the generation of a QSAR model. While incorporating some CoMFA features, it introduces the fourth dimension, which is the conformational Boltzmann sampling, and enables the method to be used as a receptor-independent (RI) method as well as receptor-dependent (RD) method in which the geometry of the receptor is known. It should be noted that their 4D-QSAR method does not solve the alignment problem but it allows a rapid evaluation of individual trial alignments [51]. Such 4D-QSAR implementation can be found in XMAP program [51,52]. Recently, it has been shown that 5D- and 6D-QSAR can be used for multiple representations of the receptor as well as its solvation states [53–55]. In the reported 5D-QSAR method, Vedani *et al.* introduced a multiple representation of induced-fit hypotheses, *i.e.*, the adaptation of the receptor binding pocket to the individual ligand topology, as the fifth dimension. In other words, they generated a family of quasi-atomistic receptor surrogates [56] which are optimized by using a genetic algorithm. The binding energy was calculated as:

(12) $${\text{E}}_{\text{binding}}\approx {\text{E}}_{\text{ligand}-\text{receptor}}-{\text{E}}_{\text{solvationligand}}-\text{T}\Delta \text{S}-{\text{E}}_{\text{internalstrain}}-{\text{E}}_{\text{inducedfit}}$$

where E_ligand-receptor_ is the force field energy of the ligand-receptor interaction, E_solvation,ligand_ is the ligand desolvation energy, TΔS is the change in the ligand entrophy upon receptor binding, E_internal strain_ is the change in ligand internal energy upon receptor binding, and E_induced fit_ is the energy uptake required for adapting the receptor surrogate [54].

The 5D-QSAR method was tested on a set of 65 *NK-1* receptor antagonists and a set of 131 *Ah* receptor ligands, achieving predictive r^2^ values of 0.837 and 0.832, while 4D-QSAR model resulted in 0.834 and 0.795, respectively [54]. They concluded that the binding affinities of new molecules were predicted more accurately with 5D-QSAR than with other lower dimension models. In the reported 6D-QSAR model, the simultaneous consideration of different solvation models was introduced by mapping parts of the surface area with different solvent properties [55]. 3D, 4D, 5D and 6D models were explored as comparison studies and the results showed the 6D-QSAR model produced the best predictive r^2^ of 0.885 [55]. Both 5D- and 6D-QSAR methods are implemented in the *Quasar* and *VirtualToxLab* software [56,57].

## 4. Comparison of 2D or Fragment-Based QSAR *versus* 3D or nD-QSAR Methods

In general, the predictive quality of 3D-QSAR methods depends on several factors such as the quality of molecular alignments/superimpositions, and information on ligand bioactive conformations. Especially molecular superimpositions are subjective and ligand bioactive conformations always remain unclear when there is no structural information on the corresponding receptor-ligand complexes. Conventional CoMFA results may often be non-reproducible because the model depends on the orientation of alignment of molecules, which can be varied and subjective. Although various improved methods and other procedures, which were discussed earlier in the paper, have been introduced to overcome major limitations of 3D-QSAR methods, *i.e.*, subjective molecular alignment and bioactive conformation problems, many of them still require manual interventions and superimpositions [58,59]. From this prospect, 2D fragment-based QSAR methods have certain advantages over multi-dimensional QSAR methods since fragment-based or 2D-QSAR methods are simple and robust and do not require subjective (or time consuming) molecular alignment or putative binding conformation or determination of 3D structures. However, the disadvantage is that some of 2D-QSAR methods such as Hansch-Fujita method may provide non-unique solutions and the overall predictive quality may not be as good as some multi-dimensional methods which are computationally more complex and demanding. A summary of QSAR methods discussed in the paper is listed in Table 1. It should be noted that the performance of each QSAR model depends on the choice of dataset and different datasets can result in different predictive q^2^ or r^2^ or SDEP values.

## 5. Conclusion

We have provided an overview of different QSAR methods and recent development in fragment-based approaches using selected studies as an illustration. Since each QSAR method has its own advantages and disadvantages, researchers should choose appropriate methods for modeling their systems. However, given a wide range of choices, it is a challenging task to pick appropriate models for one’s studies. This paper outlines many basic principles of new fragment-based QSAR methods as well as other 3D- and nD- QSAR models and illustrates some examples which may be helpful references to many researchers.

## Figures and Tables

**Table 1:** Summary of different QSAR methods and source information.

Method	nD	Dataset	Statistical model	Performance	Reference/Website
HQSAR	2D	21 Steroids	PLS	q^2^ = 0.71; r^2^ = 0.85 [11]	[11] http://www.tripos.com
FB-QSAR	2D	48 NA analogs	IDLS	r = 0.95 (r^2^ = 0.91) [19]	[19]
FS-QSAR	2D	85 bis-sulfone analogs; 83 COX2 analogs	MLR	r^2^ = 0.68; r^2^ = 0.62 [20]	[20]
TPF-QSAR	2D	282 pesticides	PM-based prediction	r^2^ = 0.75 [23]	[23]
CoMFA	3D	21 Steroids 54 HIV-1PR inhibitors	PLS	q^2^ = 0.75; r^2^ = 0.96 [11] q^2^ = 0.68; r^2^ = 0.69 [60]	[31] http://www.tripos.com [60]
CoMSIA	3D	Thermolysin inhibitors 54 HIV-1PR inhibitors	PLS	q^2^ = [0.59, 0.64] [32] q^2^ = 0.65; r^2^ = 0.73 [60]	[61,62] http://www.tripos.com [60]
Topomer CoMFA	3D	15 datasets from literature	PLS	average q^2^ = 0.636 [40]	[40] http://www.tripos.com
SOMFA	3D	31 steroids; 35 sulfonamides	MLR	r^2^ = 0.58; r^2^ = 0.53 [41]	[41]
AMSP	3D	31 steroids	MNN	q^2^ = 0.63; r^2^ = 0.67 [43]	[43]
CoMMA	3D	31 steroids	PLS	q^2^ = [0.41, 0.82] [44]	[44]
WHIM	3D	31 steroids	PCA	SDEP = 1.750 [42]	[45] http://www.vcclab.org/lab/indexhlp/whimdes.html
MS-WHIM	3D	31 steroids	PCA	SDEP = 0.742 [42]	[42]
GRIND	3D	31 steroids 175 hERG inhibitors	PLS; PCA PLS; SVM	q^2^ = 0.64; SDEP = 0.26 [47] q^2^ = 0.41; r^2^ = 0.57; SDEP = 0.72 [63]	[47] http://www.moldiscovery.com/soft_grid.php [63]
4D-QSAR	4D	20 DHFR inhibitors; 42 PGF_2_a analogs; 40 2-substituted dipyridodiazepione inhibitors 33 p38-MAPK inhibitors	PLS GL-PLS	r^2^ = [0.90, 0.95]; r^2^ = [0.73, 0.86]; r^2^ = [0.67, 0.76] [51] q^2^ = [0.67, 0.85] [64]	[51] http://www.seascapelearning.com/4DsgiSW/ [64]
5D-QSAR	5D	65 NK-1 antagonists; 131 Ah ligands	MLR	r^2^ = 0.84; r^2^ = 0.83 [54]	[54] http://www.biograf.ch
6D-QSAR	6D	106 estrogen receptor ligands	MLR	q^2^ = 0.90; r^2^ = 0.89 [55]	[55] http://www.biograf.ch
HQSAR = Hologram QSAR FB-QSAR = Fragment-based QSAR FS-QSAR = fragment-similarity-based QSAR TPF-QSAR = Top priority fragment QSAR CoMFA = Comparative molecular field analysis CoMSIA = Comparative molecular similarity indices analysis SOMFA = Self-organizing molecular field analysis AMSP = Autocorrelation of molecular surface properties CoMMA = Comparative molecular moment analysis WHIM = Weighted holistic invariant molcular QSAR MS-WHIM = Molecular surface WHIM GRIND = Grid independent descriptor				PLS = Partial least square IDLS = Iterative double least square PM = Priority matrix MNN = Multilayer neural networks MLR = Multiple linear regression PCA = Principal component analysis	q^2^ = cross-validated r^2^ SDEP = standard deviation of errors of prediction
